# The American X-ray Journal

**Published:** 1897-08

**Authors:** 


### THE AMERICAN X-RAY JOURNAL. Vol. I, No. 1. May, 1897. Heber Robarts, M. D., Editor, 2914 Morgan Street, St. Louis, Mo. Subscription price, $1 per year; single copies, 15 cents.

This is a new medical journal "devoted to the practical application of the new science and to the physical improvement of man."

It is well illustrated with photogravures relating to the subject of this new and wonderful force.

The new journal offers several premiums: *The X-Ray Journal* for one year, and a book, entitled *The A B C of the X-Rays* for $1.40. *The Journal* for one year and *The X-Ray Photography of the Invisible* for $1.40.

*The Journal* for one year and *Roentgen Rays* and *Phenomena of the Anode and Cathode* for $2.

